# Polarity‐Reversal of Exchange Bias in van der Waals FePS_3_/Fe_3_GaTe_2_ Heterostructures

**DOI:** 10.1002/advs.202409210

**Published:** 2024-11-04

**Authors:** Han Xiao, Bingbing Lyu, Mengjuan Mi, Jian Yuan, Xiandong Zhang, Lixuan Yu, Qihui Cui, Chaofan Wang, Jun Song, Mingyuan Huang, Yufeng Tian, Liang Liu, Takashi Taniguchi, Kenji Watanabe, Min Liu, Yanfeng Guo, Shanpeng Wang, Yilin Wang

**Affiliations:** ^1^ School of Integrated Circuits Shandong Technology Center of Nanodevices and Integration State Key Laboratory of Crystal Materials Shandong University Jinan 250100 China; ^2^ School of Physical Science and Technology ShanghaiTech University Shanghai 201210 China; ^3^ Shandong Wanbo Technologies Co. LTD Jinan 250100 China; ^4^ State Key Laboratory of Crystal Materials Institute of Crystal Materials Shandong University Jinan 250100 China; ^5^ Department of Physics Southern University of Science and Technology Shenzhen 518055 China; ^6^ School of Physics Shandong University Jinan 250100 China; ^7^ Research Center for Materials Nanoarchitectonics National Institute for Materials Science Tsukuba 305‐0044 Japan; ^8^ Research Center for Electronic and Optical Materials National Institute for Materials Science Tsukuba 305‐0044 Japan; ^9^ ShanghaiTech Laboratory for Topological Physics ShanghaiTech University Shanghai 201210 China

**Keywords:** exchange bias, non‐local manipulation, polarity‐reversal, van der Waals heterostructure

## Abstract

Exchange bias (EB) in antiferromagnetic (AFM)/ferromagnetic heterostructures is crucial for the advancement of spintronic devices and has attracted significant attention. The common EB effect in van der Waals heterostructures features a low blocking temperature (*T*_b_) and a single polarity. In this work, a significant EB effect with a *T*_b_ up to 150 K is observed in FePS_3_/Fe_3_GaTe_2_ heterostructures, and in particular, the EB exhibits an unusual temperature‐dependent polarity‐reversal behavior. Under a high positive field‐cooling condition (e.g., *μ_0_H* ≥ 0.5 T), a negative EB field (*H*_EB_) is observed at low temperatures, and with increasing temperature, the *H*_EB_ crosses zero at ≈20 K, subsequently becomes positive and later approaches zero again at *T*_b_. A model composed of a top FePS_3_/interfacial FePS_3_/Fe_3_GaTe_2_ sandwich structure is proposed. The charge transfer from Fe_3_GaTe_2_ to FePS_3_ at the interface induces net magnetic moments (∆*M*) in FePS_3_. The interface favors AFM coupling, and thus the reversal of ∆*M* of the interfacial FePS_3_ leads to the polarity‐reversal of EB. Moreover, the EB can be extended to the bare Fe_3_GaTe_2_ region of the Fe_3_GaTe_2_ flake partially covered by FePS_3_. This work provides opportunities for a deeper understanding of the EB effect and opens a new route toward constructing novel spintronic devices.

## Graphical abstract

A significant EB effect with temperature‐dependent polarity‐reversal behavior is observed in FePS_3_/Fe_3_GaTe_2_ heterostructures under high cooling fields (e.g., *μ_0_H* ≥ 0.5 T). A model composed of a top FePS_3_/interfacial FePS_3_/Fe_3_GaTe_2_ sandwich structure is proposed. Charge transfer from Fe_3_GaTe_2_ to FePS_3_ induces net magnetic moments (∆*M*) in FePS_3_, and the reversal of ∆*M* of interfacial FePS_3_ leads to the polarity‐reversal of EB.

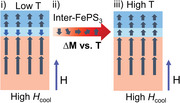

## 1 Introduction

The magnetic proximity effect, originating from the interfacial coupling of heterostructures, facilitates a plurality of intriguing physical phenomena, including skyrmions,^[^ ^1^, ^2^ ^]^ magnons,^[^ ^3^, ^4^ ^]^ exchange bias (EB),^[^ ^5^ ^]^ etc., and has garnered considerable attention. EB arises from the exchange coupling between a ferromagnetic (FM) layer and an antiferromagnetic (AFM) layer,^[^ ^6^, ^7^ ^]^ which causes the spins of the FM layer to be pinned by the AFM layer,^[^ ^8^ ^]^ or applies a torque to the AFM layer.^[^ ^9^, ^10^ ^]^ The properties of AFM and FM materials, including crystal structure, magnetic anisotropy, magnetic domains, as well as interface quality, have a significant impact on the EB effect.^[^ ^5^, ^11^ ^]^ Achieving a high‐quality interface with 3D materials is challenging due to lattice mismatch and atomic diffusion during the epitaxial growth process.^[^ ^12^ ^]^ Van der Waals (vdW) magnets facilitate to form an atomically sharp and ultraclean interface because their surfaces are free of dangling bonds,^[^ ^13^, ^14^, ^15^, ^16^ ^]^ and thus provide a desired platform for the investigation of EB.

Significant EB has been broadly observed in various vdW AFM/FM heterostructures, e.g., CrCl_3_/Fe_3_GeTe_2_,^[^ ^17^ ^]^ MnPX_3_/Fe_3_GeTe_2_ (X = S, Se),^[^ ^18^, ^19^ ^]^ CrPS_4_/Fe_3_GeTe_2_,^[^ ^20^ ^]^ CrOCl/Fe_3_GeTe_2_,^[^ ^21^ ^]^ FePS_3_/Fe_5_GeTe_2_,^[^ ^22^ ^]^ CrI_3_/MnBi_2_Te_4_,^[^ ^23^ ^]^ etc. In the FePS_3_/Fe_3_GeTe_2_ heterostructure, the Curie temperature (*T*_C_) of Fe_3_GeTe_2_ was elevated from 150 to 180 K, and the coercive field was also enhanced by ≈100%.^[^ ^24^ ^]^ The EB effect is dependent on the magnitude of the magnetic field during the cooling process. In the CrPS_4_/(Fe_0.74_Co_0.26_)_3_GeTe_2_ heterostructure, a negative EB was observed with a low cooling field, while the EB was suppressed or even eliminated with an increased cooling field.^[^ ^20^ ^]^ In oxidized‐Fe_3_GeTe_2_/Fe_3_GeTe_2_/CrSe heterostructure, a substantially large exchange bias field (*H*_EB_) of ≈90 mT was observed at low temperature, and the *H*_EB_ gradually diminishes in magnitude as temperature increases until reaching the blocking temperature (*T*_b_, the temperature at which the *H*_EB_ becomes zero).^[^ ^25^ ^]^ The EB effect can also be influenced by various extrinsic stimuli. For instance, in the FePSe_3_/Fe_3_GeTe_2_ heterostructure, modulation of interface spacing through pressure engineering significantly enhances the *H*_EB_ and *T*_b_.^[^ ^11^ ^]^ In another example, the application of a solid protonic gate in the FePS_3_/Fe_5_GeTe_2_ heterostructure toggles the presence or absence of EB by directly introducing protons into the interface.^[^ ^22^ ^]^ The *H*_EB_ is sensitive to the cooling field, interface spacing, and carrier density, while the polarity of EB does not change with varying temperatures. Polarity‐reversal of EB is valuable for the design of novel spintronic devices^[^ ^26^, ^27^ ^]^ and has been observed in certain 3D systems,^[^ ^28^, ^29^ ^]^ while it is scarce in vdW heterostructures.

Among the intrinsic vdW magnets, the recently discovered ferromagnet Fe_3_GaTe_2_ has the highest *T*_C_ (up to 380 K for bulk and 350 K for 9.5 nm nanosheet), and exhibits itinerant ferromagnetism with robust large perpendicular magnetic anisotropy, positioning it as a promising candidate for practical applications.^[^ ^30^, ^31^, ^32^, ^33^, ^34^ ^]^ The Ising‐type antiferromagnet FePS_3_ features a zigzag antiferromagnetic structure and strong out‐of‐plane magnetic anisotropy,^[^ ^35^ ^]^ and maintains a constant Néel temperature (*T*_N_) of 115 K regardless of thickness from bulk to monolayer.^[^ ^36^ ^]^ In addition, FePS_3_ has a band gap of 2.18 eV in a thin layer and behaves as an insulator,^[^ ^37^ ^]^ ensuring that current mainly flows through the metallic (or semiconducting) layer in the heterostructure through electrical measurements. Thus, the vdW heterostructure of FePS_3_/Fe_3_GaTe_2_ is appealing for exploring the EB effect.

In this work, a significant EB was observed in FePS_3_/Fe_3_GaTe_2_ heterostructures by both anomalous Hall effect (AHE) measurements and reflective magnetic circular dichroism (RMCD). Furthermore, the EB induced at the interface of the FePS_3_/Fe_3_GaTe_2_ heterostructure can laterally extend to the exposed region of the same Fe_3_GaTe_2_ flake, and such non‐local EB effect is attributed to the itinerant ferromagnetism and the single‐domain state of Fe_3_GaTe_2_. More interestingly, the EB features an unusual polarity‐reversal behavior (from negative EB to positive EB) with increasing temperature under a suitable high‐field cooling condition (e.g., *μ_0_H* ≥ 0.5 T). An intuitive model consisting of top FePS_3_/interfacial FePS_3_/Fe_3_GaTe_2_ is proposed, and the reversal of the net magnetic moments of the interfacial FePS_3_ layer is responsible for the polarity‐reversal of EB.

## 2 Results and Discussion

### 2.1 Construction of FePS_3_/Fe_3_GaTe_2_ Heterostructure

**Figure 1:**
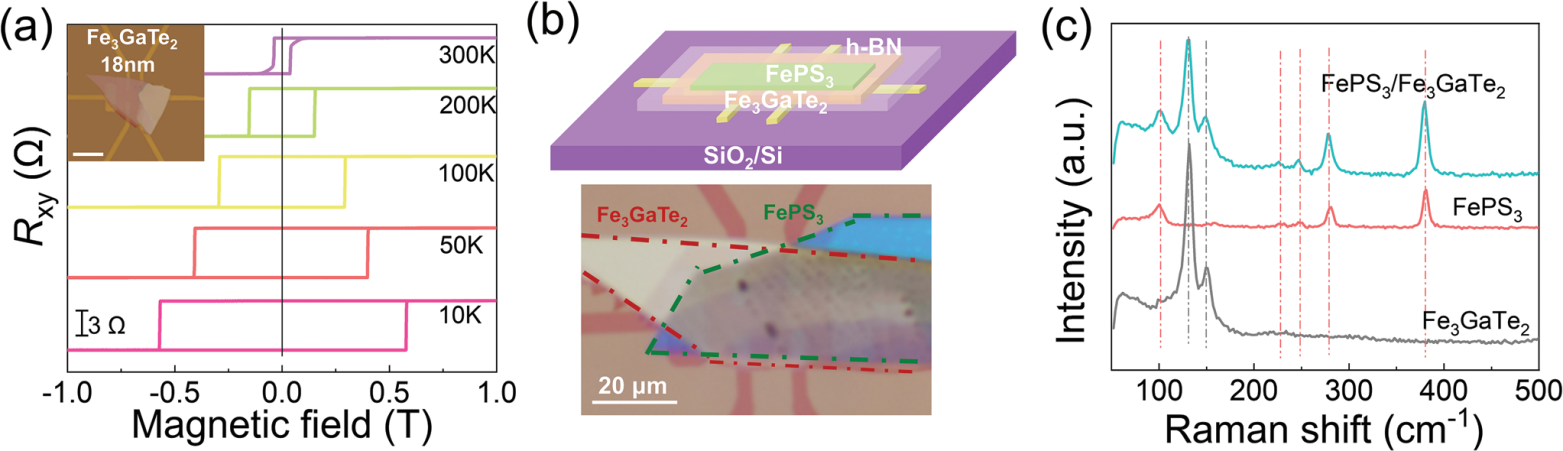
a) Temperature‐dependent *R*_xy_ for isolated Fe_3_GaTe_2_ flake (18 nm). Inset: the optical image of the isolated Fe_3_GaTe_2_ device (scale bar 20 µm). b) Schematic of the vdW FePS_3_/Fe_3_GaTe_2_ heterostructure covered with a h‐BN layer (upper panel), and optical image of Device 1 with FePS_3_ (5 nm)/Fe_3_GaTe_2_ (25 nm) heterostructure (lower panel, scale bar 20 µm). c) Raman spectra of FePS_3_/Fe_3_GaTe_2_ heterostructure, individual Fe_3_GaTe_2_ flake, and individual FePS_3_ flake.

Thin Fe_3_GaTe_2_ flakes, FePS_3_ flakes, and FePS_3_/Fe_3_GaTe_2_ heterostructures were obtained by mechanical exfoliation and dry transfer techniques in a glove box, and details can be found in the experimental sections. **Figure** **1**a illustrates anomalous Hall resistance (*R*_xy_) of Fe_3_GaTe_2_ flake with a thickness of ≈18 nm at different temperatures, indicating the absence of EB in the isolated Fe_3_GaTe_2_ flakes. The upper and lower panels in Figure 1b show a schematic diagram and an optical image of the constructed FePS_3_/Fe_3_GaTe_2_ heterostructure (Device 1), respectively, where a thin FePS_3_ layer (5 nm) is stacked on a Fe_3_GaTe_2_ layer (25 nm), and the Fe_3_GaTe_2_ layer is in direct contact with the pre‐pattered electrodes. The FePS_3_/Fe_3_GaTe_2_ heterostructure was protected by covering a h‐BN layer. Raman scattering techniques were employed to assess the quality of the FePS_3_/Fe_3_GaTe_2_ heterostructure, as shown in Figure 1c, the Raman signatures of the heterostructure region display a superposition of the Raman peaks of individual Fe_3_GaTe_2_ and FePS_3_ flakes,^[^ ^24^, ^30^, ^36^ ^]^ and no additional peaks are observed.

### 2.2 Temperature‐ and *H*_cool_‐Dependent Polarity‐Reversal of EB

**Figure 2:**
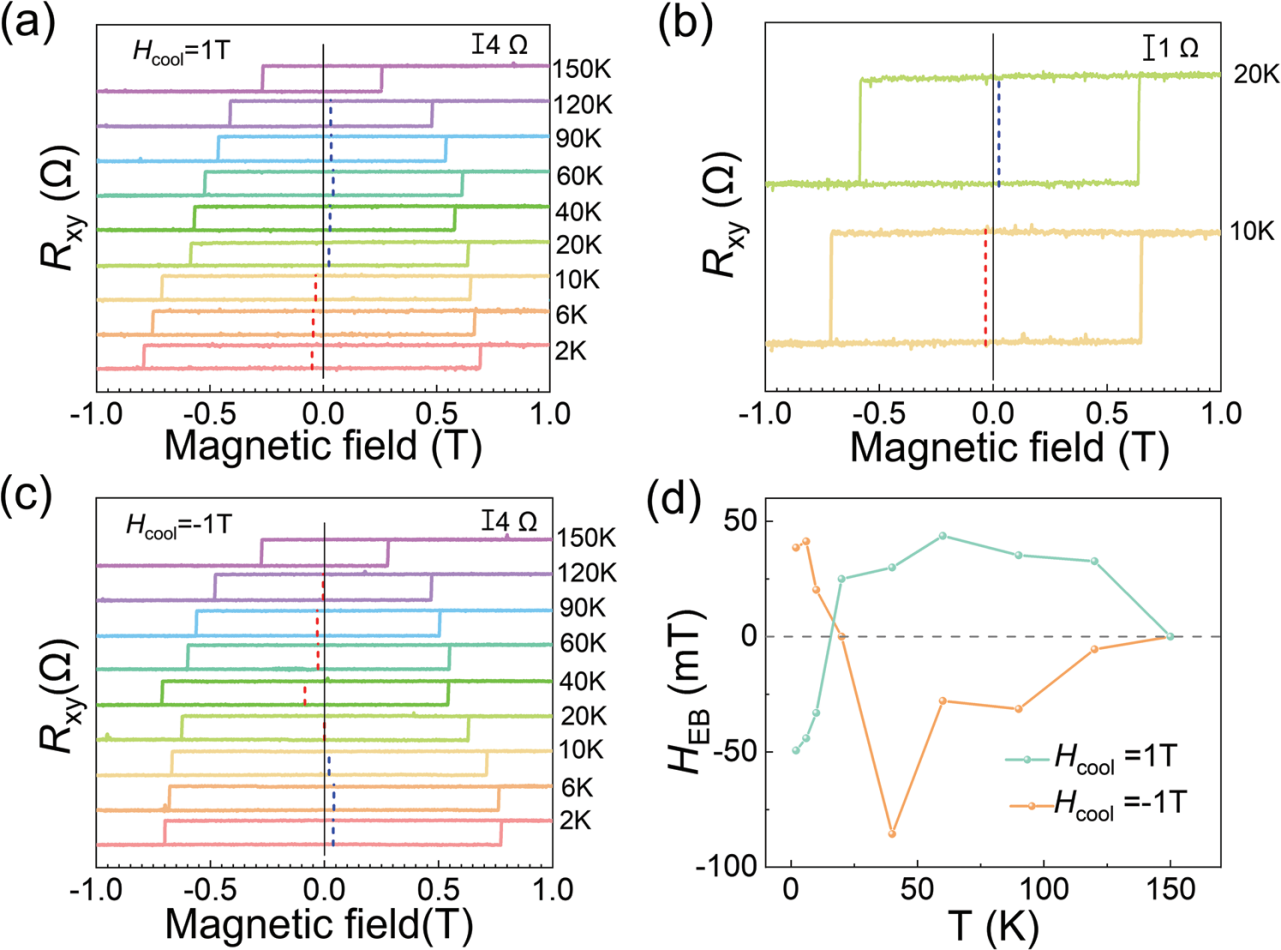
Significant EB effect in Device 1. a) Temperature‐dependent *R*_xy_ under *H*_cool_ = 1 T. b) Comparison of *R*_xy_ at 10 and 20 K under *H*_cool_ = 1 T. c) Temperature‐dependent *R*_xy_ under *H*_cool_ = −1 T. d) Temperature‐dependent *H*_EB_ under *H*_cool_ = ± 1 T.

The interfacial exchange interaction of FePS_3_/Fe_3_GaTe_2_ heterostructure was initially investigated by AHE measurement. **Figure** **2**a illustrates the typical temperature‐dependent *R*_xy_ of Device 1 with a positive cooling field (*H*_cool_) of 1 T. The *H*_cool_, perpendicular to the surface of the device, was applied at 320 K, after which the device was cooled down to a preset temperature for measurements. An obvious asymmetrical hysteresis loop with respect to the zero magnetic field was observed at low temperatures, indicating the emergence of EB. *H*_EB_, which characterizes the strength of EB in a system, is defined by a relation *H_EB_* = (*H_C_^+^* + *H_C_^−^*)/2 where *H_C_^+^* and $H_{C}^{-}$ represent the positive coercive field and negative coercive field, respectively.^[^ ^7^, ^20^ ^]^ At 2 K, the *H*_EB_ is negative, indicating that the polarity of EB is negative. As temperature increases, the *H*_EB_ crosses zero and gradually becomes positive, demonstrating that the polarity of EB reverses from negative to positive ≈20 K, and remains positive until *T*_b_ of 150 K. To establish a direct comparison, the hysteresis loops of Device 1 at 10 and 20 K are shown in Figure 2b.

When the direction of the cooling field is reversed, the sign of *H*_EB_ is also reversed. As shown in Figure 2c, when a negative *H*_cool_ of −1 T was applied, the *H*_EB_ was positive at 2 K, and as the temperature increased, the *H*_EB_ crossed zero and gradually became negative, also demonstrating the polarity‐reversal behavior of EB with increasing temperature. Figure 2d compares the temperature‐dependent *H*_EB_ with *H*_cool_ = 1 T and *H*_cool_ = −1 T. The overall trends of the temperature‐dependent *H*_EB_ for *H*_cool_ = ± 1 T are nearly symmetric, and both exhibit polarity‐reversal behavior of EB at ≈20 K. The amplitude of |*H*_EB_| exhibits a non‐monotonic relationship with temperature: |*H*_EB_| initially decreases until ≈20 K, then increases to reach a maximum value at 40–60 K, and later gradually decreases until finally disappears at ≈150 K. Moreover, the slight asymmetry observed in *H_EB_* under *H_cool_ *= ± 1 T, especially at 40 K, may be attributed to the pinning effect induced by interfacial defects formed during the fabrication of the heterostructure. The pinning effect may be dependent on specific conditions, under *H_cool_* = ± 0.5 T, the temperature‐dependent *H_EB_* appears more symmetric, as shown in Figure S2 (Supporting Information). The *T*_b_ in FePS_3_/Fe_3_GaTe_2_ heterostructure significantly surpasses that of FePS_3_/Fe_5_GeTe_2_ (*T*_b_ ≈30 K)^[^ ^22^ ^]^ and FePSe_3_/Fe_3_GeTe_2_ (*T*_b_ ≈20 K).^[^ ^11^ ^]^ The substantially high *T*_b_ is attributed to the strong magnetic coupling at the interface between the ultra‐thin FePS_3_ and Fe_3_GaTe_2_ layers, which is further demonstrated by the large |*H*_EB_| greater than 50 mT. Moreover, the *T*_b_ is higher than the *T*_N_ of FePS_3_, which may arise from possible short‐range spin correlation in FePS_3_.^[^ ^38^ ^]^

**Figure 3:**
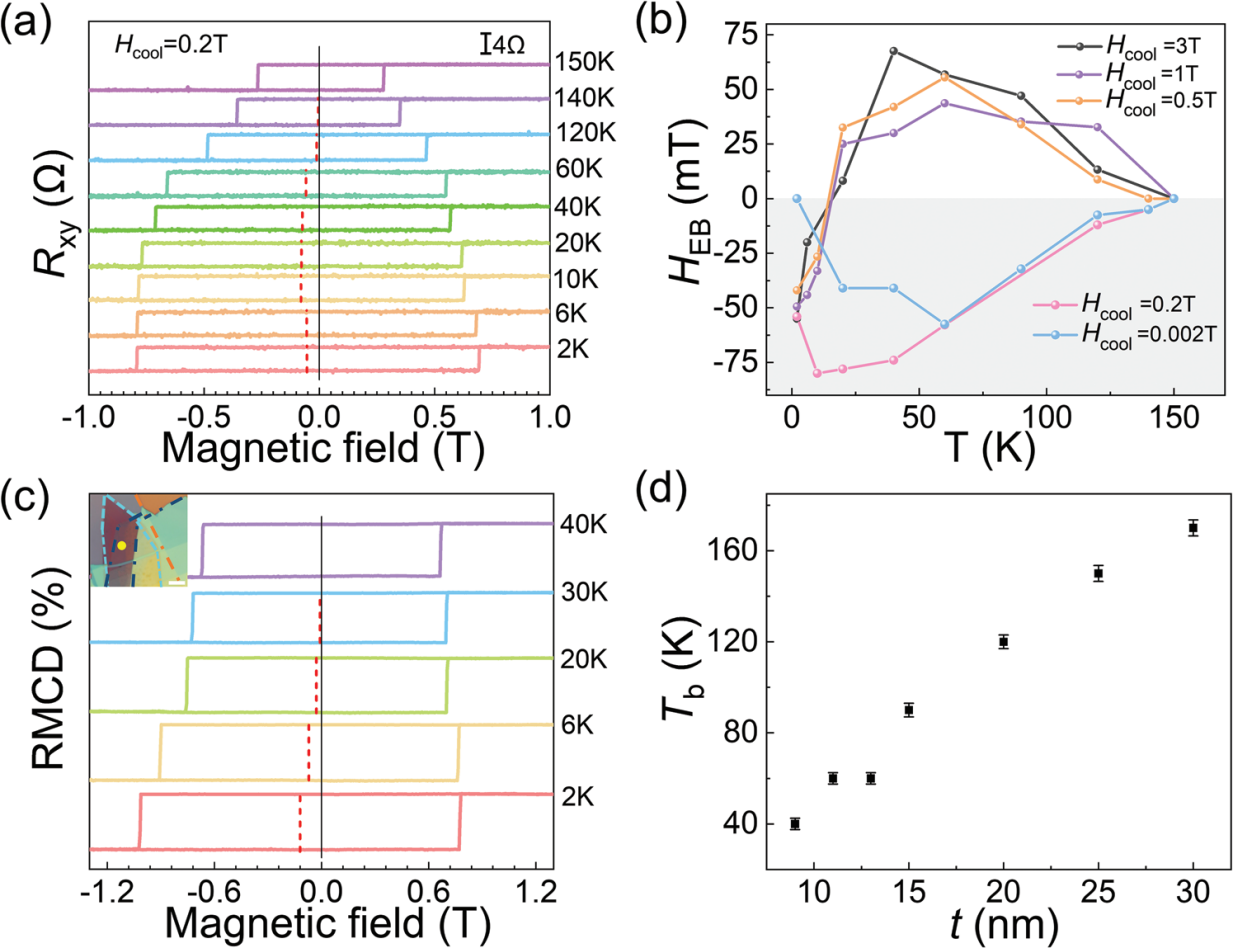
a) Temperature‐dependent *R*_xy_ for Device 1 under *H*_cool_ = 0.2 T. b) Temperature‐dependent *H*_EB_ for Device 1 under applied *H*_cool_ varied from 0.002, 0.2, 0.5, 1 and 3 T. c) Temperature‐dependent RMCD hysteresis loops of FePS_3_ (5 nm)/Fe_3_GaTe_2_ (9 nm). Inset: optical image of FePS_3_/Fe_3_GaTe_2_ heterostructure (scale bar 10 µm). d) Thickness‐dependent *T*_b_ of the FePS_3_ (5 nm)/Fe_3_GaTe_2_ (*t* nm) heterostructures. Error bars represent deviation.

According to the Meiklejohn–Bean model,^[^ ^39^ ^]^ EB is also affected by the magnitude of the cooling field. The temperature‐dependent *R_xy_* of Device 1 under *H*_cool_ varied from 0.002, 0.2, 0.5, and 3 T were further investigated. As shown in **Figure** **3**a, under *H*_cool_ = 0.2 T, the negative EB is observed and the polarity remains negative until *T*_b_ of 150 K. The non‐monotonic relationship between |*H*_EB_| and temperature is also observed, where |*H*_EB_| first increases and then decreases as the temperature increases. The temperature‐dependent *R*_xy_ of Device 1 under other cooling fields is illustrated and discussed in supporting information (Figures S3, S4, Supporting Information). Figure 3b summarizes the temperature‐dependent *H*_EB_ under different magnitudes of *H*_cool_. As the temperature increases, the polarity of EB reverses from negative to positive at ≈20 K for a suitable high cooling field of *H*_cool_ ≥ 0.5 T, while remaining negative for a relatively low cooling field of *H*_cool_ ≤ 0.2 T, evidencing that the temperature‐dependent polarity‐reversal of EB requires a suitable field‐cooling condition. In addition, the maximum values of |*H*_EB_| slightly vary depending on the magnitudes of *H*_cool_, and reach a maximum |*H*_EB_| of 76 mT for *H*_cool_ ≤ 0.2 T. The *T*_b_ is unaffected by the magnitudes of *H*_cool_, and the EB effect under varying cooling fields disappears at ≈150 K. Further, as shown in Figure S5 (Supporting Information), the similar temperature‐dependent polarity‐reversal behavior of EB was also observed in other devices with different Fe_3_GaTe_2_ thickness (30, 15, 13 and 10 nm), and the temperature at which the polarity reverses decrease as the Fe_3_GaTe_2_ thickness decreases. Therefore, it is evident that the temperature‐dependent polarity reversal of EB in FePS_3_/Fe_3_GaTe_2_ heterostructures is robust and repeatable.

The EB effect in FePS_3_/Fe_3_GaTe_2_ heterostructure was also observed by RMCD, which reveals the interfacial coupling at the microscopic scale.^[^ ^21^ ^]^ The temperature‐dependent RMCD hysteresis loops of isolated Fe_3_GaTe_2_ (10 nm) are symmetric with respect to the zero magnetic field (Figure S6, Supporting Information), revealing the absence of EB, which is also consistent with the AHE measurements. Figure 3c illustrates the temperature‐dependent RMCD hysteresis loops of the FePS_3_/Fe_3_GaTe_2_ (5 nm/9 nm) heterostructure, and the corresponding optical image is shown in the inset of Figure 3c. A negative EB with an absolute value |*H*_EB_| of 126 mT was observed at 2 K, and |*H*_EB_| gradually decreases as the temperature increases and becomes zero until *T*_b_ of 40 K. Moreover, because of the zero field‐cooling condition for RMCD measurements, the polarity‐reversal behavior of EB was not observed.

Figure 3d summarizes the variation of *T*_b_ as a function of the Fe_3_GaTe_2_ thickness. The *T*_b_ monotonically increases from 40 to 170 K as the FM layer thickness increases from 9 to 30 nm with the FePS_3_ thickness fixed at 5 nm. The maximum absolute value of *H_EB_* also monotonically increases from ≈38 to ≈120 mT with increasing the Fe_3_GaTe_2_ layer thickness, as shown in Figure S7 (Supporting Information). Such a similar relationship between the strength of EB and FM layer thickness was also observed in other heterostructures such as CrCl_3_/Fe_3_GeTe_2_,^[^ ^17^ ^]^ CrPS_4_/(Fe_0.74_Co_0.26_)_3_GeTe_2_^[^ ^20^ ^]^ and oxidized‐Fe_3_GeTe_2_/Fe_3_GeTe_2_.^[^ ^40^ ^]^ The enhancement of EB as the FM layer thickness increases is attributed to that the FM flakes with larger thickness have higher volume magnetization^[^ ^17^, ^20^, ^40^ ^]^ and favor improved interface quality due to the reduction of surface roughness of the FM layer.^[^ ^41^, ^42^ ^]^

### 2.3 Non‐Local EB Effect

**Figure 4:**
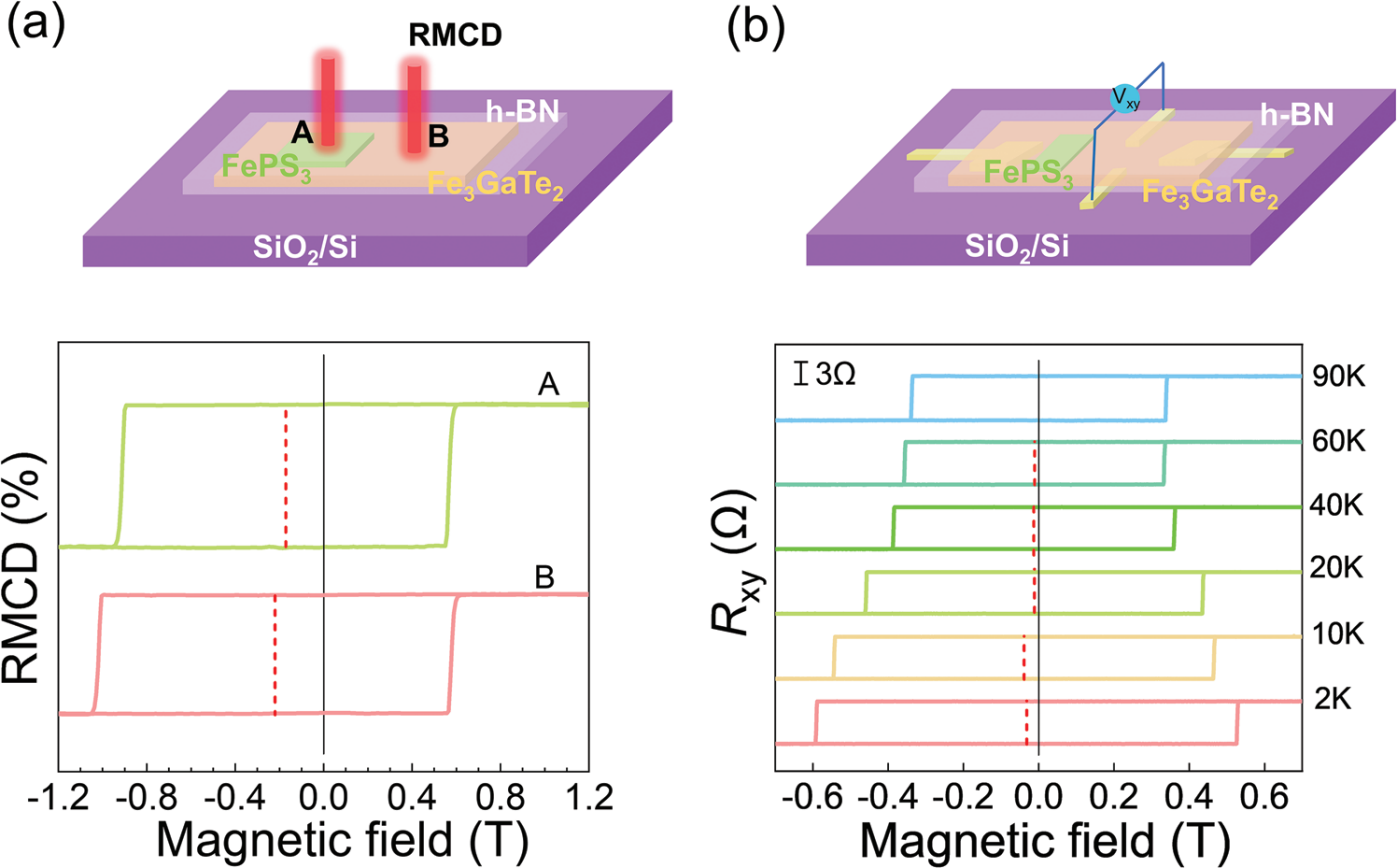
a) Schematic of the half‐covered FePS_3_/Fe_3_GaTe_2_ heterostructure measured by RMCD (upper panel). RMCD hysteresis loops of FePS_3_/Fe_3_GaTe_2_ region (region A) and exposed Fe_3_GaTe_2_ region (region B) at 2 K (lower panel). b) Schematic of the half‐covered FePS_3_/Fe_3_GaTe_2_ device measured by AHE (upper panel). Temperature‐dependent *R*_xy_ of exposed Fe_3_GaTe_2_ region under *H_cool_* = 0.5 T, which is connected to the region of the same Fe_3_GaTe_2_ flake (15 nm) covered by FePS_3_ (5 nm) in Device 2 (lower panel).

Particularly, the non‐local EB effect was observed in FePS_3_/Fe_3_GaTe_2_ heterostructures. The FePS_3_/Fe_3_GaTe_2_ heterostructures with varied interfacial configurations were constructed, where the interfacial configuration is adjusted by selectively exposing or covering part of the Fe_3_GaTe_2_ flakes, as illustrated in the upper panels of **Figures** **4**a,b. As shown in Figure 4a, the RMCD was conducted on two separated regions (labeled A and B in Figure 4a) of the same Fe_3_GaTe_2_ flake, region A was directly covered by FePS_3_ flake while region B was exposed. RMCD hysteresis loops of both A and B regions exhibit similar features, where the negative EB and a substantially large |*H*_EB_| even up to 200 mT at 2 K were observed. In addition, the temperature‐dependent *R*_xy_ measured on a bare region of the Fe_3_GaTe_2_ flake (Device 2, the other region of the same Fe_3_GaTe_2_ flake was covered by FePS_3_) also reveals the non‐local EB effect. As shown in the lower panel of Figure 4b, a negative EB, a substantially large |*H*_EB_| up to 40 mT, and a *T*_b_ of ≈90 K were observed. The *T_b_* and negative EB of Device 2 with Fe_3_GaTe_2_ partially covered by FePS_3_ are consistent with those of Device 4 with Fe_3_GaTe_2_ having a similar thickness and fully covered by FePS_3_ (Figure S8, Supporting Information). Such phenomena indicate that EB can effectively propagate in the transverse direction at the micrometer scale. Manipulating magnetization at lateral scales provides a promising way for constructing novel spin logic devices such as magnetoelectric spin‐orbit logic (MESO).^[^ ^43^ ^]^ The non‐local EB effect is attributed to the itinerant ferromagnetic properties and the single magnetic domain of Fe_3_GaTe_2_ flakes.^[^ ^11^, ^44^ ^]^

### 2.4 Physical Mechanism of Polarity‐Reversal of EB

**Figure 5:**
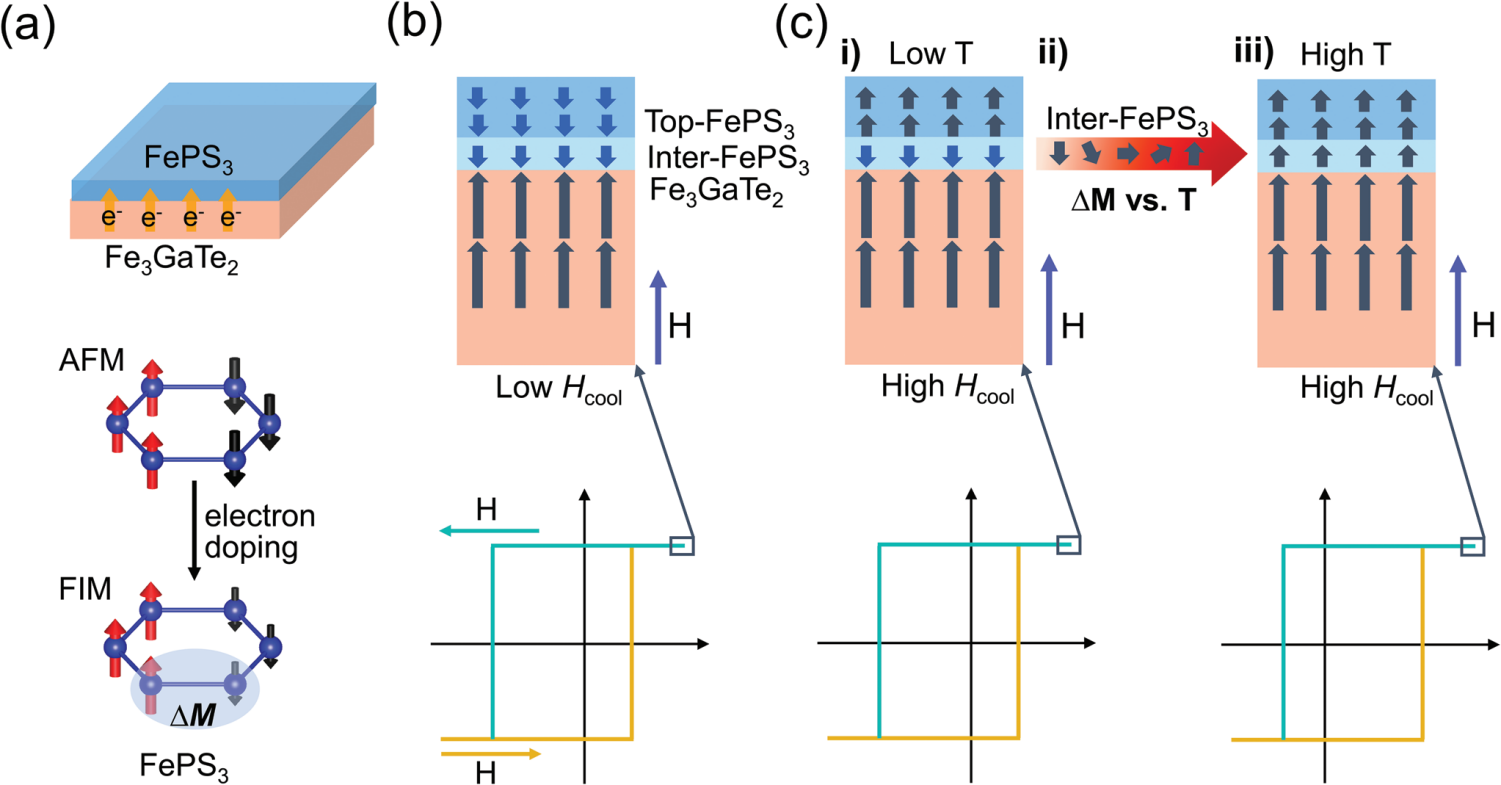
a) Upper panel: schematic of electron transfer from Fe_3_GaTe_2_ to FePS_3_ at the interface. Lower panel: the zigzag AFM order of FePS_3_ and the FIM order with ∆*M* of doped FePS_3_, where arrows indicate the orientation and magnitude of the magnetic moments of Fe atoms. b,c) Upper panels: schematic of a model for the magnetic configurations of top‐FePS_3_/inter‐FePS_3_/Fe_3_GaTe_2_ under low *H*_cool_ b) and high *H*_cool_ c), respectively. Arrows indicate the orientation and magnitude of interfacial Fe_3_GaTe_2_ or ∆*M* of inter‐FePS_3_ and top‐FePS_3_. Lower panels: negative EB under low *H*_cool_, negative EB under high *H*_cool_ at a low temperature c‐i), and positive EB under high *H*_cool_ at a high temperature (c‐iii), respectively. (c‐ii) Schematic diagram showing the upward rotation of ∆*M* of the inter‐FePS_3_ as temperature increases.

The observed polarity‐reversal of EB as the temperature increases under a suitable high *H*_cool_ is intriguing and rarely reported in other vdW heterostructures. For FePS_3_/Fe_3_GaTe_2_ heterostructure, FePS_3_ possesses a higher work function (4.9 eV, insulating)^[^ ^45^ ^]^ compared to Fe_3_GaTe_2_ (≈4.4 eV, metallic),^[^ ^46^, ^47^ ^]^ such that charge transfer from Fe_3_GaTe_2_ to FePS_3_ occurs at the interface, as illustrated in the upper panel of **Figure** **5**a. Electron doping causes the magnetic ground state of FePS_3_ to transform from AFM order to ferrimagnetic (FIM) order, as demonstrated by the intercalation of organic cations in FePS_3_.^[^ ^48^ ^]^ In the FIM order of electron‐doped FePS_3_, the two ferromagnetic zigzag chains of Fe atoms possess unequal magnetic moments, producing net magnetic moments (∆*M*), as shown in the lower panel of Figure 5a. The FIM‐FePS_3_ retains the large coercive field of FePS_3_, which prevents ∆*M* from being fully aligned within the sweeping magnetic field range during EB measurements at low temperatures, such that an unusual temperature‐dependent hysteresis loop of FIM‐FePS_3_ was observed.^[^ ^48^ ^]^ As shown in Figure S9 (Supporting Information), as temperature increases to 60 K, the hysteresis loops become more pronounced, and ∆*M* gradually increases, which is ascribed to that thermal kinetic energy promotes the alignment of magnetic moments. Thermal fluctuations would weaken the exchange coupling as the temperature increases, therefore the trade‐off between the magnitude of ∆*M* and thermal fluctuations lead to the maximum EB in a range of 40–60 K as the temperature increases.

The mechanisms of the negative EB under low *H*_cool_ and temperature‐dependent polarity‐reversal (from negative to positive) of EB under high *H*_cool_ were then discussed. If the interfacial exchange coupling is FM, a negative EB is always formed regardless of the field‐cooling conditions. If the interfacial exchange coupling is AFM, the polarity of EB is dependent on the field‐cooling conditions.^[^ ^7^, ^49^ ^]^ Therefore, the observation of positive EB under high *H*_cool_ in FePS_3_/Fe_3_GaTe_2_ heterostructures indicates that their interfacial exchange coupling is AFM.

Moreover, the temperature‐dependent polarity‐reversal of EB cannot be explained by a simple AFM coupling interface, and a relatively complex model composed of multiple structures is necessary to understand such phenomena. As shown in Figure 5b, the FePS_3_/Fe_3_GaTe_2_ heterostructure can be divided into three portions^[^ ^19^, ^50^ ^]^: the bottom Fe_3_GaTe_2_ portion, the interfacial FePS_3_ portion in direct contact with Fe_3_GaTe_2_ (inter‐FePS_3_), and the top FePS_3_ portion (top‐FePS_3_). The magnetic configurations of the FePS_3_/Fe_3_GaTe_2_ heterostructure are determined by the competition between the energy of the interfacial exchange coupling at the interfacial‐FePS_3_/Fe_3_GaTe_2_ interface (*E*_ex_), the interlayer magnetic interaction between the top‐FePS_3_ and inter‐FePS_3_ portions of doped FePS_3_ layer (*E*_inter_), and the Zeeman energy for aligning the magnetic moments (*E*_Z_).^[^ ^8^ ^]^ Take the heterostructure being field‐cooled along a positive direction as an example. The *E*_Z_ tends to make the magnetic moments of Fe_3_GaTe_2_ and the ∆*M* of both inter‐FePS_3_ and top‐FePS_3_ point to the direction of the external magnetic field (upward); the *E_e_*_x_ tends to make the ∆*M* of inter‐FePS_3_ point to a direction (downward) opposite to that of the magnetic moments of Fe_3_GaTe_2_ due to the interfacial AFM coupling; and the *E*_inter_ tends to make the ∆*M* of inter‐FePS_3_ and the ∆*M* of top‐FePS_3_ align in the same direction.

When *H*_cool_ is low, *E*_Z_ is substantially small, causing the ∆*M* of inter‐FePS_3_ to point downward driven by the *E*_ex_, and the ∆*M* of top‐FePS_3_ to point downward driven by the *E*_inter_, such that the corresponding magnetic configuration of the top‐FePS_3_/inter‐FePS_3_/Fe_3_GaTe_2_ is initially formed as ↓↓/↓↓/↑↑, as illustrated in the upper panel of Figure 5b. To overcome the AFM interfacial coupling, a larger magnitude of *H_C_* ^‐^ $H_{C}^{-}$ is required to reverse the magnetic moments of Fe_3_GaTe_2_ during the backward process, while a smaller magnitude of *H_C_* ^+^ $H_{C}^{+}$ is needed during the forward process, and thus a negative EB is observed, as shown in the lower panel of Figure 5b.

When enhancing *H*_cool_ to a high level, *E*_Z_ increases, and the ∆*M* of top‐FePS_3_ points upward driven by the *E*_Z_, while the direction of ∆*M* of the inter‐FePS_3_ is determined by the competition between *E*_ex_, *E*_Z_, and *E*_inter_. At low temperatures, *E*_ex_ dominates the magnetic configuration of the inter‐FePS_3_ (*E*_ex_ > *E*_Z_ +* E*_inter_), and thus the ∆*M* of inter‐FePS_3_ point downward, and the corresponding magnetic configuration of the top‐FePS_3_/inter‐FePS_3_/Fe_3_GaTe_2_ is initially formed as ↑↑/↓↓/↑↑, as illustrated in the upper panel of Figure 5c (i). Because the exchange interactions are short‐range interactions, the magnetic configuration of inter‐FePS_3_ dominates the EB effect. In view of this, similar to the case of the heterostructure cooled with a low *H*_cool_, a negative EB is observed, as shown in the lower panel of Figure 5c (i). As the temperature increases, thermal fluctuation increases and the *E*_ex_ decreases, the *E*_ex_ is no longer sufficient enough to pin the ∆*M* of inter‐FePS_3_ pointing downward, and the ∆*M* of inter‐FePS_3_ gradually rotates upward driven by the *E*_Z_ and *E*_inter_, as illustrated in Figure 5c (ii). Therefore, at a suitable high temperature, the ∆*M* of inter‐FePS_3_ points upward, and the corresponding magnetic configuration of the top‐FePS_3_/inter‐FePS_3_/Fe_3_GaTe_2_ is formed as ↑↑/↑↑/↑↑, as illustrated in the upper panel of Figure 5c (iii). Benefiting from AFM interfacial coupling, a smaller magnitude of *H_C_^‐^* $H_{C}^{-}$ is required to reverse the magnetic moments of Fe_3_GaTe_2_ during the backward process, while a larger magnitude of *H_C_* ^+^ $H_{C}^{+}$ is needed during the forward process, and thus a positive *H*_EB_ shown in the lower panel of Figure 5c (iii) is observed. Micromagnetic simulations based on the proposed sandwich structure were also conducted to simulate the process of gradually rotating *∆M* in the inter‐FePS_3_ layer as temperature increases under the competition between *E_inter_* and *E_ex_*, as shown in Figure S10 (Supporting Information). The results of micromagnetic simulations reveal the possible reversal of ∆*M* of the inter‐FePS_3_ as the temperature increases, which leads to the temperature‐dependent polarity reversal of EB.

## 3 Conclusion

In summary, an obvious and considerable EB effect was observed in the FePS_3_/Fe_3_GaTe_2_ heterostructure, and in particular, the EB effect exhibits non‐local properties and temperature‐dependent polarity‐reversal behavior. Due to the itinerant ferromagnetism and single magnetic domain state of Fe_3_GaTe_2_, for the Fe_3_GaTe_2_ flake partially covered with FePS_3_, the bare region exhibits similar EB characteristics as the covered region. The temperature‐dependent polarity‐reversal behavior of EB is cooling field dependent, where with increasing temperature, the negative EB reverses to the positive EB under suitable high cooling field conditions, while the negative polarity of EB remains unchanged under relatively low cooling field conditions. An intuitive model composed of top‐FePS_3_/inter‐FePS_3_/Fe_3_GaTe_2_ sandwich structure is proposed to understand the polarity‐reversal behavior, where the charge transfer from Fe_3_GaTe_2_ to FePS_3_ at the interface induces ∆*M* in FePS_3_, and the orientations of ∆*M* of the interfacial FePS_3_ layer, which is determined by the competition between *E*_ex_, *E*_inter_ and *E*_Z_, dominates the polarity of EB. The findings of this work provide new insights for understanding the EB effect in various vdW heterostructures and offer emerging strategies for constructing innovative spin logic devices.

## 4 Experimental Section

### Device Fabrication

Fe_3_GaTe_2_ and FePS_3_ bulk crystals were grown by the flux method. To minimize the oxidation and contamination, thin Fe_3_GaTe_2_ and FePS_3_ flakes were obtained by mechanical exfoliation, and the heterostructures were prepared by dry transfer techniques with poly(dimethylsiloxane) (PDMS) stamps onto the pre‐pattered electrodes in a glove box filled with Ar gas, where H_2_O and O_2_ levels were maintained below 0.1 ppm. Before performing characterization and measurements, the devices were encapsulated by h‐BN.

### Transport Measurements and RMCD Measurements

Electrical transport measurements were performed in a commercial physical property measurement system (PPMS, Quantum Design Dynacool‐9) with lock‐in techniques (SR830). The RMCD measurements were conducted using a standard lab‐made RMCD setup and the wavelength of HeNe laser was 633 nm.

### Characterization

Raman measurements were performed via an inVia confocal Raman microscope (Renishaw) with a 532 nm laser at room temperature. The laser power was kept below 0.05 mW to avoid local heating. Atomic force microscopy (AFM) images were carried out by a Benyuan system (CSPM5500). The thickness was determined by AFM and color contrast analysis based on optical images.

## Conflict of Interest

The authors declare no conflict of interest.

## Supporting information



Supporting Information

## Data Availability

The data that support the findings of this study are available from the corresponding author upon reasonable request.
