# Supplementary material for: Polarity‐Reversal of Exchange Bias in van der Waals FePS3/Fe3GaTe2 Heterostructures
Source: Adv Sci (Weinh). 2024 Nov 4;11(48):2409210. doi: 10.1002/advs.202409210 (PMC11672248; doi:10.1002/advs.202409210)
Supplement: Supplementary file 1 — Supporting Information [file ADVS-11-2409210-s001.docx]

Supporting Information

Polarity-reversal of Exchange Bias in van der Waals FePS_3_/Fe_3_GaTe_2_ Heterostructures

*Han Xiao,^1^*^#^ *Bingbing Lyu,^1^*^#^ *Mengjuan Mi, ^1^ Jian Yuan,^2^ Xiandong Zhang,^3^ Lixuan Yu,^1^ Qihui Cui,^4^ Chaofan Wang,^5^ Jun Song,^3^ Mingyuan Huang,^5^ Yufeng Tian,^6^ Liang Liu,^6^ Takashi Taniguchi,^7^ Kenji Watanabe,^8^ Min Liu,^1*^ Yanfeng Guo,^2,9*^ Shanpeng Wang,^4*^ and Yilin Wang^1*^*

^1^School of Integrated Circuits, Shandong Technology Center of Nanodevices and Integration, State Key Laboratory of Crystal Materials, Shandong University, Jinan 250100, China

^2^School of Physical Science and Technology, ShanghaiTech University, Shanghai 201210, China

^3^Shandong Wanbo Technologies Co., LTD, Jinan 250100, China

^4^State Key Laboratory of Crystal Materials, Institute of Crystal Materials, Shandong University, Jinan 250100, China

^5^Department of Physics, Southern University of Science and Technology, Shenzhen, 518055, China

^6^School of Physics, Shandong University, Jinan 250100, China

^7^Research Center for Materials Nanoarchitectonics, National Institute for Materials Science, Tsukuba, 305-0044, Japan

^8^Research Center for Electronic and Optical Materials, National Institute for Materials Science, Tsukuba, 305-0044, Japan

^9^ShanghaiTech Laboratory for Topological Physics, ShanghaiTech University, Shanghai 201210, China

*Authors to whom correspondence should be addressed: [liumin@sdu.edu.cn](mailto:liumin@sdu.edu.cn); [guoyf@shanghaitech.edu.cn](mailto:guoyf@shanghaitech.edu.cn); [wshp@sdu.edu.cn](mailto:wshp@sdu.edu.cn); [yilinwang@email.sdu.edu.cn](mailto:yilinwang@email.sdu.edu.cn)

^#^These Authors contributed equally to this work


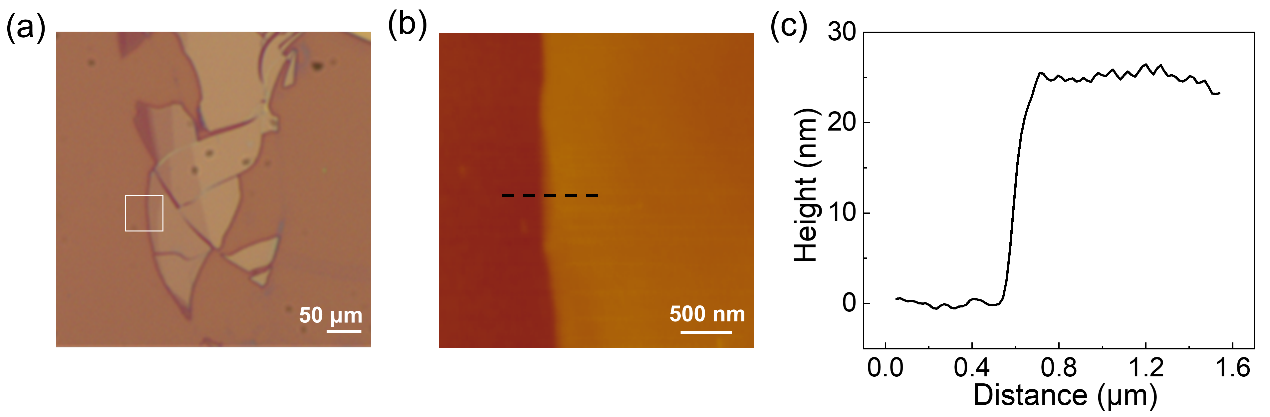


**Figure S1**. a) Optical image of Fe_3_GaTe_2_ flake with a thickness comparable to the Fe_3_GaTe_2_ flake in Device 1. b) Atomic force microscopy image of the area marked by the square in (a). c) The thickness line profile of the Fe_3_GaTe_2_ flake along the dark line in (b).


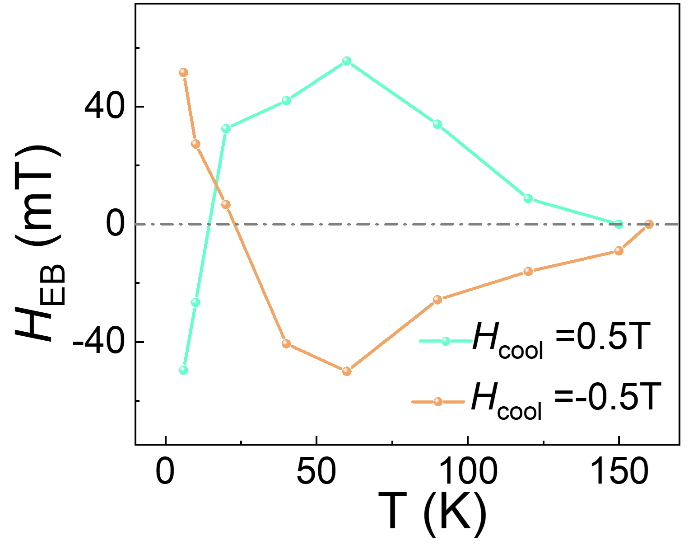


**Figure S2.** Temperature-dependent *H_EB_* for Device 1 under *H_cool_* = ± 0.5 T.


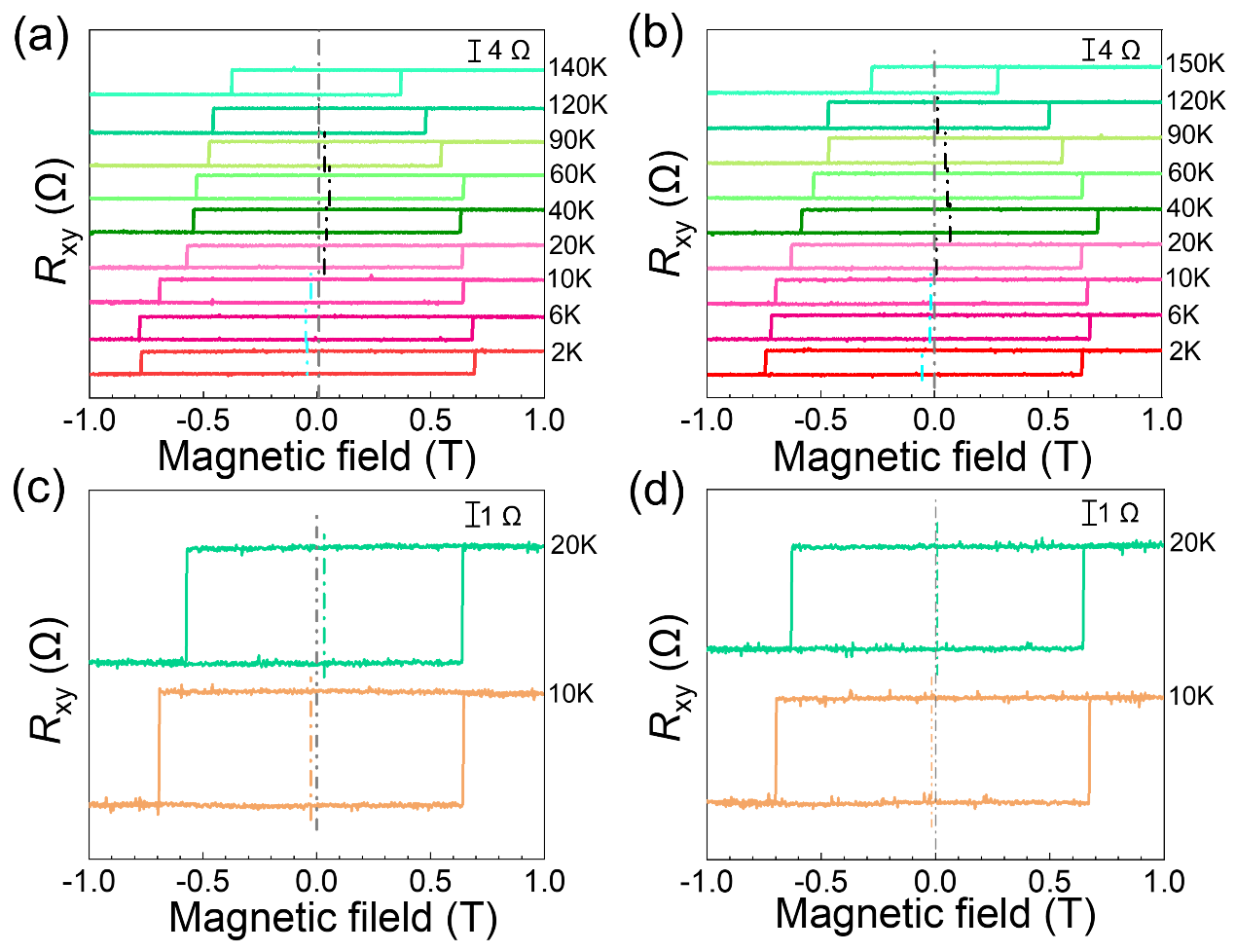


**Figure S3.** a-b) Temperature-dependent *R*_xy_ for Device 1 under *H*_cool_ of 0.5 T (a) and 3 T (b), respectively. c-d) Comparison of *R*_xy_ for Device 1 at 10 K and 20 K under *H*_cool_ of 0.5 T (c) and 3 T (d), respectively.


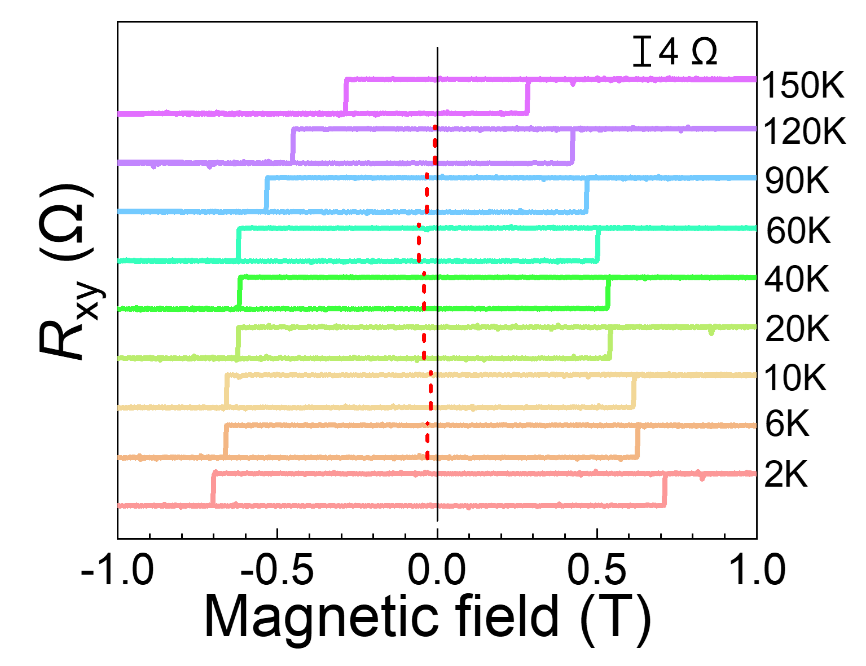


**Figure S4.** Temperature-dependent *R*_xy_ for Device 1 under *H*_cool_ = 0.002 T.


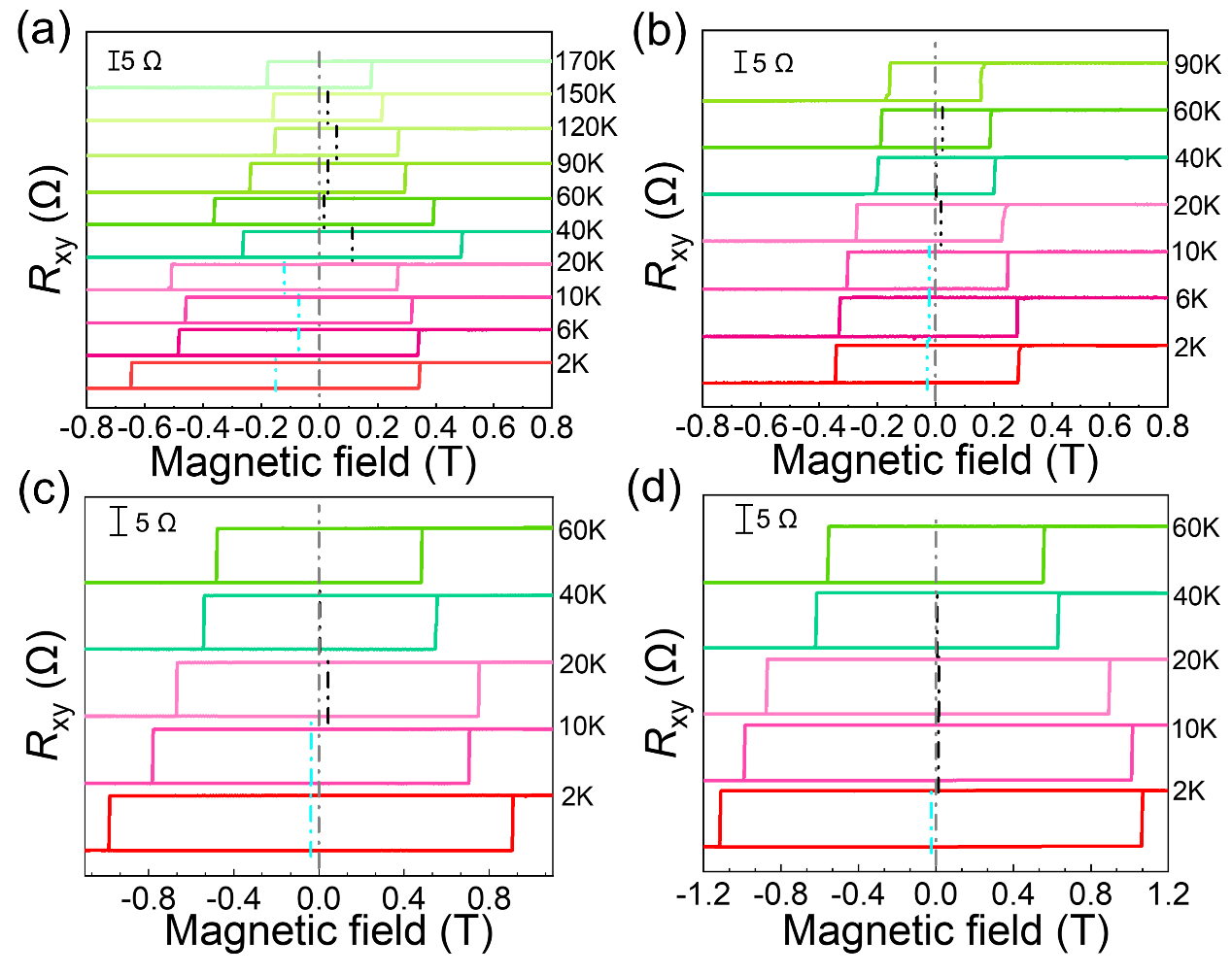


**Figure S5.** Polarity-reversal of EB effect in various Devices with the FePS_3_ thickness fixed at 5 nm. Temperature-dependent *R*_xy_ for Device 3 with 30 nm-thick Fe_3_GaTe_2_ flake under *H*_cool_ = 0.8 T (a), Device 4 with 15 nm-thick Fe_3_GaTe_2_ flake under *H*_cool_ = 0.8 T (b), Device 5 with 13 nm-thick Fe_3_GaTe_2_ flake under *H*_cool_ = 1 T (c), and Device 6 with 10 nm-thick Fe_3_GaTe_2_ flake under *H*_cool_ = 1 T (d).


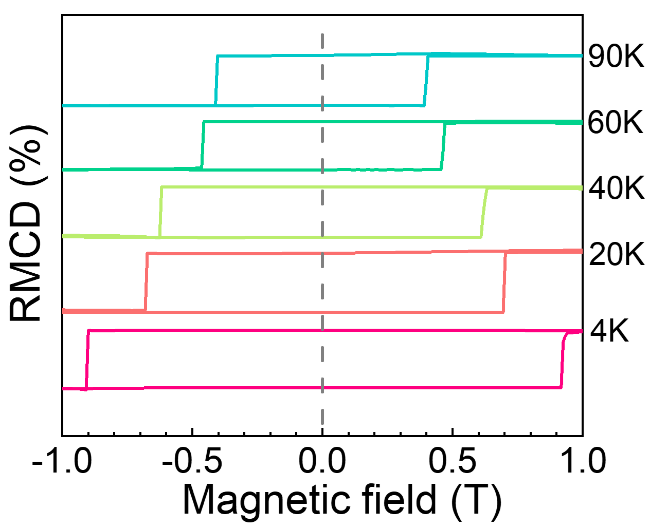


**Figure S6**. Temperature-dependent RMCD hysteresis loops of isolated Fe_3_GaTe_2_ (10 nm).


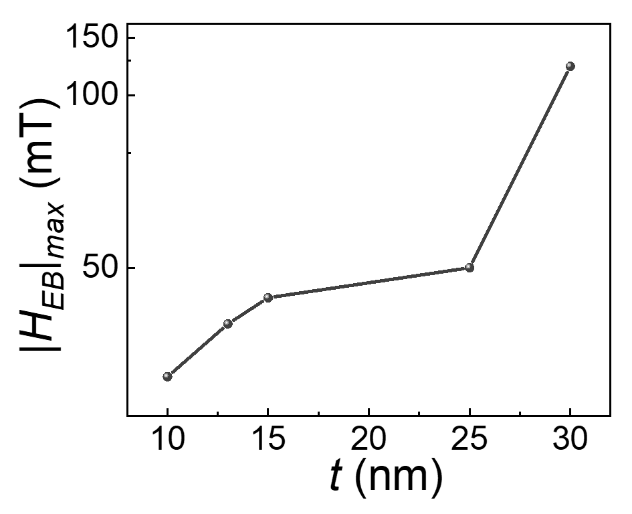


**Figure S7.** Thickness-dependent *|H_EB_|_max_* of the FePS_3_ (5 nm)/Fe_3_GaTe_2_ (*t* nm) heterostructures.


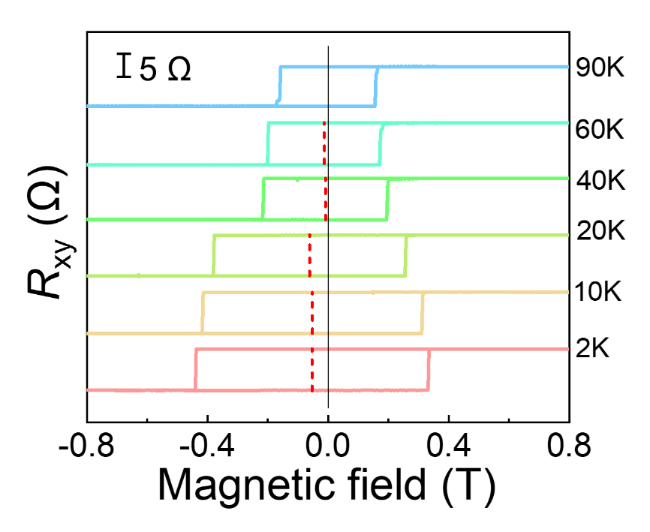


**Figure S8.** Temperature-dependent *R*_xy_ for Device 4 under *H*_cool_ = 0.5 T.

**The magnetic properties of electron-doped FePS_3_**

In FePS_3_/Fe_3_GaTe_2_ heterostructure, charge transfer occurs at the interface from Fe_3_GaTe_2_ to FePS_3_, leading to a transition of the magnetic ground state of FePS_3_ from AFM order to ferrimagnetic (FIM) order, and inducing net magnetic moments (∆*M*) in FePS_3_ layer (Figure 5a). The properties of ∆*M*, which can be reflected by the hysteresis loop of electron-doped FePS_3_, play a crucial role in determining the properties of EB. Considering the insulating nature of FePS_3_, the magnetic properties of electron-doped FePS_3_ were investigated through the field-dependent magnetization (M-H) measurements on the bulk FePS_3_ crystal intercalated with tetraheptylammonium (THA^+^) cations. The intercalation of THA^+^ cations lead to electron doping of FePS_3_, which may represent the FIM properties of electron-doped FePS_3_.^[1]^

**Figure S9** illustrates the hysteresis loops (M-H) of FIM-FePS_3_. As temperature increases to 60 K, the hysteresis loops become more pronounced: the area of the hysteresis loop increases, and the remanent magnetization also increases. Such phenomenon is contrast to the expectations where the hysteresis loop decreases monotonically with increasing temperature. Such unusual features of the hysteresis loops of FIM-FePS_3_ are attributed to the extremely large coercive field ($H_{C}$ = 38 T) of FePS_3_,^[2]^ and the FIM-FePS_3_ retains the significant coercive field. The large coercive field prevents the ∆*M* of the FIM-FePS_3_ from being fully aligned by the applied magnetic field in our measurements at low temperature. As the temperature increases, thermal kinetic energy promotes the alignment of magnetic moments parallel to the direction of the magnetic field. The trade-off between thermal fluctuations and Zeeman energy results in the more pronounced hysteresis loop as the temperature increases.


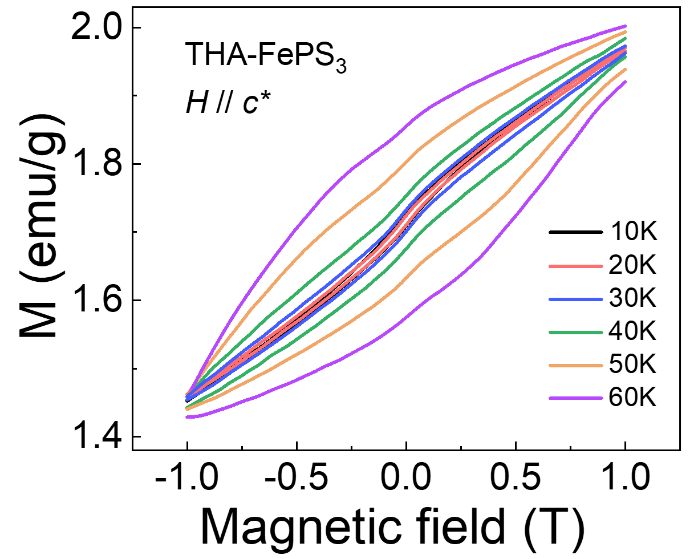


**Figure S9.** Field-dependent magnetization (M-H) of intercalated THA-FePS_3_ under a magnetic field H // c* from 10 K to 60 K.

Therefore, the magnitude of *∆M* of FIM-FePS_3_ first increases as temperature increases from low temperature. The increased *∆M* enhances the pinning effect on the Fe_3_GaTe_2_ layer, and thus enhances the strength of EB effect. Thermal fluctuations would weaken the exchange coupling as the temperature increases, therefore the trade-off between the magnitude of *∆M* and thermal fluctuations leads to the maximum EB in a range of 40 -60 K as the temperature increases. With further increasing temperature, thermal perturbations are dominated and also results in a decrease in the magnitude of *∆M*.

**Micromagnetic simulations**

To further validate the mechanism beneath the switchable EB, numerical simulations were done with the anisotropic Heisenberg model:

$$H_{EB}=\sum_{ij} J_{ij}\boldsymbol{m}_{i}\cdot\boldsymbol{m}_{j}+\sum_{i} K_{i}\left( m_{i}^{z} \right)^{2}$$

in which $\boldsymbol{m}_{i}$ is the normalized *i*^th^ magnetic moment, $J_{ij}$ represents the exchange coupling between atomic magnetic moments, $K_{i}$ denotes the perpendicular magnetic anisotropy.

As discussed in section 2.4 in main text, a three-layer magnetic structure is established, including the top-FePS_3_, inter-FePS_3_ and Fe_3_GaTe_2_ layers. Each layer contains 64×64×1 cells and periodic boundary condition is employed for in-plane directions. In top-FePS_3_ and inter-FePS_3_ layers, a pair of anti-paralleled magnetic moments is set to mimic the anti-ferromagnetism. In Fe_3_GaTe_2_ layer, one magnetic moment per cell is set to represent the net magnetization coupled to inter-FePS_3_. The effective interfacial exchange couplings for top-FePS_3_/inter-FePS_3_ and inter-FePS_3_/Fe_3_GaTe_2_ are set to the ferromagnetic and anti-ferromagnetic types.

**Figure S10** shows the temperature evolution of the magnetic structure. At low temperature, the magnetic moment of inter-FePS_3_ is oriented to -z (Figure S10a), indicating that the anti-ferromagnetic type of inter-FePS_3_/Fe_3_GaTe_2_ is favored. As temperature exceeds 30 K, inter-FePS_3_ switches to the +z magnetization, revealing the ferromagnetic type of top-FePS_3_/inter-FePS_3_ is favored at high temperature. For temperatures beyond *T_N_* = 115 K, the magnetic moments approach zero. Figure S10b displays the exchange energies of the two interfaces. At low temperature, the magnitude of exchange energy on inter-FePS_3_/Fe_3_GaTe_2_ is larger than that on top-FePS_3_/inter- FePS_3_. Therefore, the anti-ferromagnetic inter-FePS_3_/Fe_3_GaTe_2_ dominates the equilibrated magnetic states. As temperature increases, the exchange energy of inter-FePS_3_/Fe_3_GaTe_2_ quickly shrinks, and becomes smaller than the exchange energy in top-FePS_3_/inter-FePS_3_ after 30 K. Therefore, the ferromagnetic top-FePS_3_/inter-FePS_3_ dominates the equilibrated magnetic states at high temperature. At very high temperature, both interfaces exhibit negligible exchange energy due to the thermal fluctuations. Because the orientation of inter-FePS_3_ determines the polarity of EB, the above results reveal the possible polarity-switching of EB induced by thermal effects, in line with the experiments.


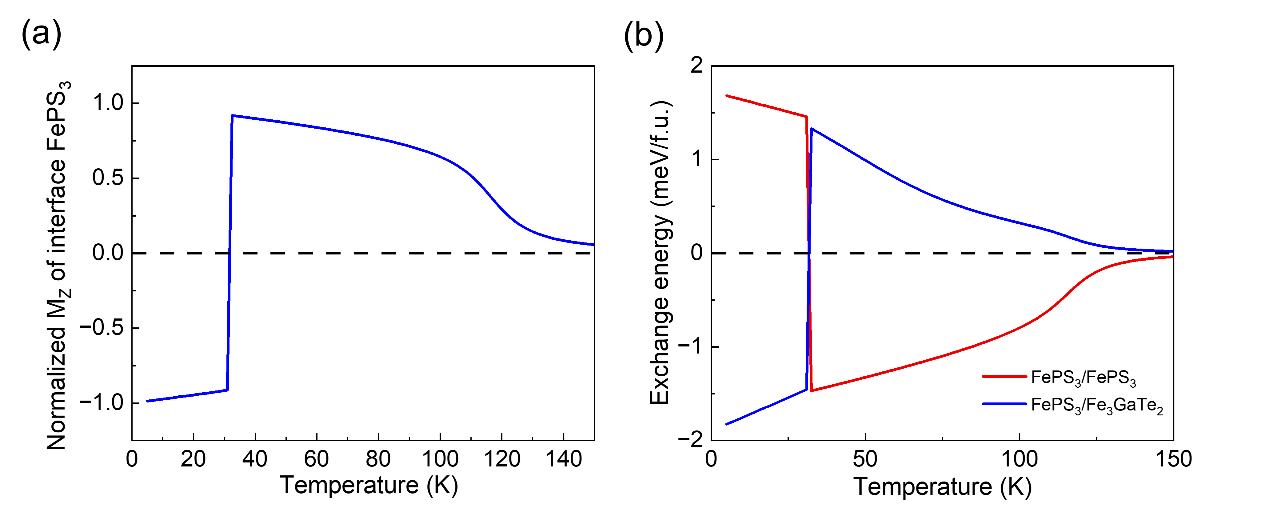


**Figure S10.** Temperature evolutions of magnetic energy and moments revealed by micromagnetic simulation. a) Evolutions of normalized z-component of magnetization of inter-FePS_3_. b) Evolutions of exchange coupling energies of top-FePS_3_/inter-FePS_3_ and inter-FePS_3_/Fe_3_GaTe_2_.

**Calculation details**

The temperature dependent evolutions of magnetizations are obtained by the Monte Carlo simulations on Heisenberg model as implemented in MCSOLVER.^[3-4]^ 2×10^5^ Metropolis sampling sweeps were used for thermal equilibrations and subsequent 2×10^7^ sweeps were conducted for the statistics on thermal quantities. The effective intra-layer exchange couplings of FePS_3_ are set to 12 meV to reproduce the *T_N_* = 115 K, and the effective intra-layer exchange couplings of Fe_3_GaTe_2_ are set to 0.9 meV. The effective interfacial exchange couplings for top-FePS_3_/inter-FePS_3_ and inter-FePS_3_/Fe_3_GaTe_2_ are set to -1.7 meV and 1 meV, corresponding to the ferromagnetic and anti-ferromagnetic couplings. Anisotropic constants are set to -1 meV to capture the strong perpendicular magnetism.

References

[1] M.-J. Mi, L.-X. Yu, H. Xiao, B.-B. Lü, Y.-L. Wang, *Acta. Phys. Sin.* **2024**, *73*, 057501.

[2] A. R. Wildes, D. Lançon, M. K. Chan, F. Weickert, N. Harrison, V. Simonet, M. E. Zhitomirsky, M. V. Gvozdikova, T. Ziman, H. M. Rønnow, *Phys. Rev. B* **2020**, *101*, 024415.

[3] L. Liu, X. Ren, J. Xie, B. Cheng, W. Liu, T. An, H. Qin, J. Hu, *Appl. Surf. Sci.* **2019**, *480*, 300.

[4] L. Liu, S. Chen, Z. Lin, X. Zhang, *J. Phys. Chem. Lett.* **2020**, *11*, 7893.
